# Health information needs of professional nurses required at the point of care

**DOI:** 10.4102/curationis.v38i1.1432

**Published:** 2015-06-11

**Authors:** Esmeralda Ricks, Wilma ten Ham

**Affiliations:** 1 Department of Nursing Science, Nelson Mandela Metropolitan University, South Africa

## Abstract

**Background:**

Professional nurses work in dynamic environments and need to keep up to date with relevant information for practice in nursing to render quality patient care. Keeping up to date with current information is often challenging because of heavy workload, diverse information needs and the accessibility of the required information at the point of care.

**Objectives:**

The aim of the study was to explore and describe the information needs of professional nurses at the point of care in order to make recommendations to stakeholders to develop a mobile library accessible by means of smart phones when needed.

**Method:**

The researcher utilised a quantitative, descriptive survey design to conduct this study. The target population comprised 757 professional nurses employed at a state hospital. Simple random sampling was used to select a sample of the wards, units and departments for inclusion in the study. A convenience sample of 250 participants was selected. Two hundred and fifty structured self-administered questionnaires were distributed amongst the participants. Descriptive statistics were used to analyse the data.

**Results:**

A total of 136 completed questionnaires were returned. The findings highlighted the types and accessible sources of information. Information needs of professional nurses were identified such as: extremely drug-resistant tuberculosis, multi-drug-resistant tuberculosis, HIV, antiretrovirals and all chronic lifestyle diseases.

**Conclusion:**

This study has enabled the researcher to identify the information needs required by professional nurses at the point of care to enhance the delivery of patient care. The research results were used to develop a mobile library that could be accessed by professional nurses.

## Introduction

Professional nurses promote the recovery or maintain the health of their patients by completing certain tasks during patient care. The tasks performed by professional nurses can be either routine or non-routine. The non-routine tasks are normally unfamiliar and require professional nurses to seek additional information for the effective completion of these tasks.

Professional nurses therefore have a need for information that is accessible, good quality, up-to-date, manageable and relevant, as well as information services that assist nurses in finding that information (Bertulis & Cheeseborough 2008; Lundgrén-Laine *et al.* 2013; Younger 2010). Furthermore, to address the nurses’ health information needs and enhance quality of treatment, clinical and public health information should be accessible and the nurses should be able to consult the relevant medical texts, guidelines and tools at the point of care.

### Background and literature review

According to Willmer (2007), the 21st century is both an information and knowledge age. There is currently an explosion of health-related knowledge and nurses are experiencing challenges in regularly accessing the most current information ‘because of being task-driven and coping with heavy workloads’ (Doran *et al.* 2007). The aforementioned authors also highlight similar findings of a study conducted by MacIntosh-Murray and Choo (2005) which reveal that nurses do not give attention to or recognise their potential information needs and knowledge gaps because of their heavy workloads and the fact that they are task-driven (Doran *et al.* 2007).

According to Breimaier, Halfens and Lohrmann (2011) nurses need ‘to demonstrate evidence to underpin practice and show that their practice is effective, efficient and worthwhile’. The World Health Organization (2002) and the International Council of Nurses (ICN 2006), respectively, stipulate that nurses have a pivotal role to play in the health system in order to meet the set health targets. They are also the major role players in determining and implementing acceptable standards of clinical nursing practice. Pressure is therefore being placed on nurses at all levels to use research findings in daily nursing practice as a basis for decision making. ‘Despite the availability of increasingly research-based information with the potential to improve nursing care quality in several fields of nursing …, nurses often fail to incorporate current research findings in their practices’ (Breimaier *et al.* 2011:1745). Therefore, a considerable gap can be detected between what is known in research and what happens in practice (Breimaier *et al.* 2011:1745). Information needs arise when persons recognise a gap in their state of knowledge and wish to resolve that anomaly (Nicholas 1996, cited in Xu *et al.* 2005).

Murphy *et al.* (2006) cite Alper *et al*. (2004), who indicate that ‘627.5 hours per month, or about 29 hours per weekday’ would be needed to maintain currency with relevant literature in primary care. The volume of information associated with keeping up-to-date evidence is frequently cited as a barrier amongst health care professionals. Information-seeking behaviours of physicians are better documented than nurses (Murphy *et al.* 2006). According to Ndosi and Newell (2010):

[*n*]urses prefer to consult human sources such as nursing colleagues, the team leader, a physician or the pharmacist because their information needs are triggered by patient needs which require quick decision-making at the point of care.

Although several studies have reported on the health information needs of patients, few studies have been conducted on the health information needs of nurses. Furthermore, studies reviewed by Xu *et al.* (2005:839) revealed ‘that nurses have unmet information needs while delivering care to patients’. The aforementioned authors also found that only a few authors reported studies pertaining to nurses’ information needs whilst there were several studies (Forsythe *et al*. 1992; Gorman 1995; Ely *et al.* 2000; Allen *et al*. 2003, all cited in Xu *et al.* 2005) that reported on the information needs of physicians.

Studies found regarding the health information needs of nurses revealed limited access to information and a high demand for value-added information services that help nurses to find good quality, up-to-date, manageable and relevant evidence (Bertulis & Cheeseborough 2008; Lundgrén-Laine *et al.* 2013; Younger 2010).

### Problem statement

Professional nurses work in dynamic environments and need to keep up to date with relevant information for current practice in nursing because keeping abreast with information is fundamental to rendering quality patient care based on evidence and improving time and cost effectiveness. Keeping up to date with current information is often challenging for professional nurses because of their heavy workload, diverse information needs and the accessibility of the required information at the point of care.

This is especially challenging for professional nurses in South Africa as there is a heavy workload because of a shortage of staff and lack of resources, specifically at the educational level (National Department of Health 2013). For example, nurses do not have access to libraries other than the hospital library, which often has a limited access to books/literature and no internet access.

With this in mind, the research questions that underpinned this study included:

- To what information do professional nurses currently have access?
- What are professional nurses’ main sources of information?
- How is the information that is currently being accessed by professional nurses utilised in practice?
- What information do professional nurses need at the point of care to enhance nursing practice in the delivery of patient care?

#### Aims of the study

The aim of the study was to explore and describe the information needs of professional nurses at the point of care that could enhance nursing practice in the delivery of patient care. The research findings were used to make recommendations to stakeholders who utilised the information to develop a mobile library that could be accessible when needed at the point of care.

#### Definition of key concepts

**Information needs:** An information need is defined by Miranda and Tarapanoff (2008) as ‘a state or process started when one perceives that there is a gap between the information and knowledge available to solve a problem and the actual solution of the problem’. For the purpose of the study, the information need will refer to the identified knowledge gap as indicated by the participants.

**Professional nurses:** A professional nurse refers to a person registered as such in terms of section 31 of the *Nursing Act*, 2005 (Act No. 33 of 2005) which stipulates that no person may practise as a professional nurse unless he or she is licensed to practise as such by the South African Nursing Council (Republic of South Africa 2006). Thus, a professional nurse refers to an individual who has graduated from an accredited basic programme for professional nurses and is employed at the research site.

**Point of care:** The concept ‘point of care’ refers to the location at which patient services are delivered (Miller-Keane & O’Toole, 2005). In this study, the point of care refers to the patient's bedside, where the study is conducted.

**Access to information:** Access to information refers to an individual's right to obtain information collected or generated by others (Definitions.net 2012). Access to information also means an individual, which is in this study the professional nurse, who has access to information that is necessary to carry out work-related activities.

**Information sources:** Information sources are the various means by which a person is informed about something or knowledge is provided or shared with someone, a group of people or an organisation. In this study, information sources are referred to as people, organisations, speeches, documents, pictures or observations and could be in either print or non-print formats.

#### Significance of work

The findings of this study with regard to identifying the health information needs of professional nurses at the point of care in order to enhance the delivery of patient care, were used to develop a mobile library that could be accessed by professional nurses whenever the need arose. Accessing information at the point of care facilitates sound clinical decision making by professional nurses which could enhance patient care.

## Research design

### Research approach and method

The researcher utilised a quantitative, descriptive survey design to conduct this study. Watson *et al.* (2008) state that quantitative research is based on numerical data or quantities and is concerned with the examination of aggregated views, whilst descriptive research is used to describe (Terre Blanche, Durrheim & Painter 2006).

A quantitative design was selected in order to quantify the results and to generalise the findings regarding the information needs of professional nurses at the point of care that could enhance nursing practice in the delivery of patient care.

#### Population and sampling

The target population comprised 757 professional nurses employed at the state hospital in various wards, units and departments. A statistician was consulted regarding the sampling techniques and size of the sample. A list of all the wards, units and departments was obtained from one of the Nursing Directors. Simple random sampling was used to select a sample of the wards and units. For example, if there were four medical wards, then the four wards were numbered and one was selected randomly by simple random sampling. The same sampling technique was used for units and departments. Once the wards, units and departments were selected, a convenience sample of 250 participants was selected from the randomly-selected wards units and departments for inclusion in the study.

#### Data collection

The researcher gained access into the field by requesting permission from the relevant authorities and thereafter making arrangements with the nursing service managers with regard to suitable dates and times for data collection. Two hundred and fifty structured self-administered questionnaires were distributed amongst the participants. The questionnaire comprised both open- and closed-ended questions and requested data about professional nurses’ current access to information, sources of information, application of information accessed and information needs at the point of care. The data collection was executed by the researcher and two fieldworkers who were trained prior to data collection.

#### Data treatment

The data were processed and captured by the researcher using an Excel spreadsheet. Descriptive statistics were used to analyse and describe the data. Tables and figures were used to display the research data using percentages and averages. The researcher conducted a pre-test of the instrument in the same manner as the actual study amongst 25 participants conveniently selected from the various wards, units and departments to establish the reliability and validity of the choice of research design and methods. No changes were made to the questionnaire after the pilot study, the results of which were not included in the final data analysis.

#### Context of the study

This study was conducted at a state hospital in an urban setting where information technology support was available to the professional nurses if they encountered problems using smart phones for accessing information at the point of care.

## Results

Two hundred and fifty questionnaires were distributed by the principle researcher and two additional fieldworkers to the various participants. Only 136 questionnaires were completed and returned, resulting in a 54.4% response rate.

### Demographic data analysis

The majority of the participants (*n* = 91; 66.6 %) was between 46 and 65 years of age and 82.4% (*n* = 112) of the participants were women. The majority of the participants (*n* = 71; 52.2%) were qualified as a general nurse and midwife, whilst 34.6% (*n* = 47) of the participants indicated that they completed the four-year integrated nursing qualification comprising General, Midwifery, Community and Psychiatric Nursing Science.

### Current access to information

The questions posed in this section of the questionnaire elicited information to establish whether the participants currently have access to information; what health related topics they are currently accessing; whether they are satisfied with the information received; the problems experienced in accessing information; and how much time is spent on reading the accessed information.

### Access to information

A total of 92 (67.6%) participants indicated that they had access to some information whilst 20.6% (*n* = 28) indicated that they did not have any access to information. The other 11.8% (*n* = 16) of the participants did not respond to the question.

### Topics currently being accessed

A total of 112 (82%) participants indicated that they needed and accessed information on the following topics whilst caring for patients within the previous month (Table 1).

**TABLE 1:** Information needed and accessed within the previous month (*n* = 112).

Information required and accessed within the previous month	Percentage
Tuberculosis	26.8
HIV	26.8
Diabetes mellitus	14.3
Hypertension	10.7
Asthma	9.8
Poisoning and drug overdose	9.8
Epilepsy	8.9
Cardiovascular disease	8.9
Oncology	8
Neurology	6.3
Chest infections and pneumonia	5.4
Malnutrition and Kwashiorkor	5.4
Arthritis	4.5
Diarrhoea and vomiting	4.5
Eye conditions	3.6
Meningitis	3.6
Malaria	2.7
Eclampsia	2.7
Spinal injuries	2.7
Tonsillectomy	2.7
Teenage pregnancy	2.7
Pelvic inflammatory disease	2.7
Other: allergies, snake bite, peripheral neuropathy, endometriosis, Tetrology of Follet, venous ulcers, peptic ulcers, cholecystitis, haemophilia, colporrhaphy, cerebrovascular accident, aplasia, Hirchsprung's Disease, appendicitis, haemangioma, inguinal hernia, orchiopexy, ectopic pregnancy, fibroids, laryngectomy, mechanical ventilation and spontaneous abortion	39.3

### Satisfaction with information received

Slightly more participants (*n* = 58; 43%) indicated that they were not satisfied with the health information they received than the participants who indicated that they were satisfied (*n* = 45; 33%). The reasons cited for the dissatisfaction indicated that the information received was inadequate and a need was expressed for information on a wider range of topics. The remaining participants (*n* = 33; 24%) did not respond to the question.

### Problems experienced in accessing information

When searching for information, the majority of the participants (*n* = 58; 42.6%) indicated that they are too busy and do not find the time to search for information, whilst a significant third (*n* = 45; 33.1%) of the participants indicated that the journals are too expensive to purchase. Even though there are numerous education resources available, the participants were not aware of the open access journals. A further 25% (*n* = 34) of the participants indicated that information was inaccessible to them.

### Amount of time spent reading information accessed

The majority of the participants (*n* = 83; 61%) indicated that they spend between 15 minutes to more than one hour per week reading information, whilst 29% (*n* = 39) of the participants did not respond to this question and 5% (*n* = 7) of the participants indicated that they had no time to read.

### Current information sources accessed

The following table reflects the participants’ current sources of information and the frequency with which they access these sources (Table 2).

**TABLE 2:** Participants’ current sources of information and the frequency with which they access the sources (*n* = 136).

Information sources	Frequency in accessing information from the sources					
		**Daily (%)**	**Weekly (%)**	**Monthly (%)**	**Quarterly (%)**	**Other (%)**	**Total (%)**
Electronic (internet, CD ROM)	1.8.	2.9	5.1	1.8	1.8	13.4
Colleagues/peers/friends	42.6	12.5	4.4	0.7	1.8	62.0
Supervisors	36	16.2	12.5	0	1.5	66.2
Doctors	40.4	6.6	5.9	0.7	1.8	55.4
Peer-reviewed nursing/medical journals	9.6	1.8	36.8	8.8	3.7	61.7
Radio	35.3	8.1	5.1	0	5.9	54.3
Books	1.8	11.8	9.6	0.7	4.4	28.3
Other	5.1	1.5	0.7	0	1.5	8.8

### Ways in which information is being used by participants

**FIGURE 1:**
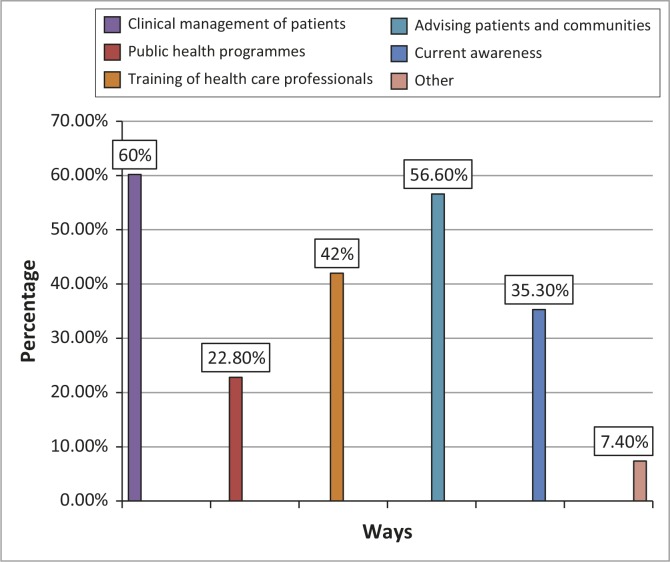
Ways in which current accessed information is used by professional nurses in practice.

The participants were presented with various options with regard to accessing information in practice; they were allowed to select more than one (Figure 1).

### Information needs of professional nurses required at the point of care

This section of the questionnaire focused on questions to elicit information that would indicate whether the participants experienced knowledge gaps; the information that the participants considered a requirement at the point of care; and the preferred frequency and length of information that they wish to access.

### Participants who experienced gaps in their knowledge

The results reflect that almost half (*n* = 60; 44%) of the participants indicated that they experienced gaps in their knowledge, whilst 39% (*n* = 53) of the participants indicated that they did not. The remaining 17% (*n* = 23) of the participants did not respond.

### Information that the participants considered a requirement at the point of care

The participants indicated that they were mainly interested in obtaining information with regard to the treatment (*n* = 107; 79%), prevention and control measures (*n* = 107; 79%), clinical presentation (*n* = 106; 78%) and the disease management protocols related to various disease topics. Table 3 below reflects the various disease topics for which the participants required the aforementioned information at the point of care.

**TABLE 3:** Information identified as a requirement at the point of care (*n* = 136).

Health related topics	Frequency (%)	Number response (%)	Total
Extensively drug-resistant tuberculosis	76.4	23.6	100
HIV	72.8	27.2	100
Multi-drug-resistant tuberculosis	72.1	27.9	100
Antiretroviral drugs	71.3	28.7	100
Tuberculosis	60.3	39.7	100
Prevention of mother-to-child transmission of HIV	53	47	100
Non-communicable disease e.g. Cardiovascular, Diabetes Mellitus	52.2	47.8	100
Sexually-transmitted infections	43.4	56.6	100
Malaria	42.6	57.4	100
Public/community health legislation	40.4	59.6	100
Laboratory services	39.7	60.3	100
Disease surveillance	38.2	61.8	100
Research abstracts on topical subjects			
in health	38.2	61.8	100
Immunisable diseases	36.8	63.2	100
Diarrhoeal diseases	33.8	66.2	100
Maternal health issues in general	33.8	66.2	100
Family planning	29.4	70.6	100

### Preferred frequency and length of information accessed

Most of the participants (*n* = 92; 68%) indicated that they would like to receive health-related information on a monthly basis, whilst a small percentage (*n* = 23; 17%) of the participants indicated that they would like to receive information on a weekly basis.

The results reflect that 52% (*n* = 71) of the participants preferred three or more printed pages on the information that they required so that they could use the information for teaching and learning. The number of pages indicated that participants preferred articles which were short and to the point instead of lengthy articles. More than a quarter of the participants (*n* = 38; 28%) indicated that they preferred one printed page of information which was concise and quick to read and absorb. A few (*n* = 14; 10%) of the participants indicated that too much information was not important as long, as they could access the information that they required.

## Ethical considerations

Ethical behaviour is important in research, as in any other field of human activity. The principles underlying research ethics are universal and concern issues such as honesty and respect for the rights of individuals (Welman, Kruger & Mitchell 2010:181).

The research proposal was submitted to the local university's Faculty of Health Sciences, Faculty Research, Technology and Innovative Committee for approval and also to the Research Ethics Committee (Human). Meetings were held with partners involved in this research project and permission was granted by the Chief Executive Officer of the identified state hospital to conduct this research (reference number H08-HEA-NUR-003).

### Potential benefits and hazards

There are no known potential hazards. The stakeholders might not have benefited directly from the study; however, insight into the information needs of professional nurses required at the point of care provided the researcher with data which guided the development of recommendations to stakeholders to develop a mobile library via smart phones which will be made accessible to improve health outcomes and provide better care.

The ethical principles of beneficence, respect for human dignity and the principle of justice were observed throughout this study in order to protect participants from any harm.

### Recruitment procedures

All staff on duty at the time of data collection were approached and only staff members who agreed to participate in the study were included.

### Informed consent

Informed consent was obtained from participants prior to completion of the questionnaires by means of a consent form and by explaining to them the purpose and objectives of the study.

### Data protection

The questionnaires did not request names or names of organisations and participants completing the questionnaire remained anonymous. Completed questionnaires were locked in a special data cupboard at the Department of Nursing Science for a period of five years.

## Trustworthiness

### Reliability and validity

Reliability and validity were ensured throughout the research process. Validity was enhanced by contextualising and adapting a structured self-administered questionnaire that was used by AED-SATTELIFE in previous studies of this nature for data collection. AED-SATTELIFE is a Boston-based, non-profit organisation that suggested that the researcher use the questionnaire. Reliability was enhanced as a pre-test of the instrument was conducted to eliminate any ambiguities with regard to the questionnaire, data collection and the data analysis processes. Content validity and face validity was ensured as a literature review was conducted and experts in various fields were involved to verify the content of the questionnaire and the technical aspects of the research. The research proposal was submitted to the Faculty of Health Sciences’ Faculty Research, Technology and Innovative Committee and the university's Research Ethics Committee (Human) for approval. Strict ethical principles were adhered to throughout the research process.

## Discussion

### Outline of the results

The results of the study will be discussed as follows:

#### Demographic data analysis

The results of this study reflected that the majority of the participants were middle-aged women. The results are similar to the overall workforce profile in South Africa which reflects very few men and a large proportion of nurses approaching retirement whilst fewer younger nurses enter the field. This finding is significant as many of the people of this age group do not have computer literacy skills and have not done a computer literacy course; the findings should be considered to overcome barriers in the access of information.

#### Current access to information

The majority of the participants indicated that they had access to some form of information whilst less than a quarter of the participants indicated that they did not have any access to information. The latter could be a reason for concern as healthcare and nursing professions are dynamic and ever changing. Furthermore, one needs to have access to information in order to keep abreast of these changes. The onus rests with the registered nurses to keep themselves updated with current information, because one of the eight main concepts that constitute the core of the professional conduct of registered nurses and midwives is that they ‘remain professionally competent and abreast of the latest developments in the health area in which [*they*] function in accordance with his/her scope of practice’ (National Department of Health 2013).

Many of the participants indicated that they needed and accessed information on a wide range of topics whilst caring for their patients within the previous month. The most common diseases for which information was needed in the previous month were tuberculosis (TB) and HIV, followed by the chronic diseases related to lifestyle. The latter results are an indication of the disease profile of South Africa (National Department of Health 2012).

Even though many participants indicated that they had access to a wide range of information, the research results revealed that more than two-fifths of the participants were dissatisfied with the information that they accessed because of its limited scope and the fact that it did not meet their needs. Some participants indicated that they did not find the time to search for information because of their busy work schedule and 15 minutes of reading new information appeared to be the maximum time spent by most of the participants.

#### Professional nurses**’** main sources of information

It is evident from the findings of this study that professional nurses used humans (supervisors, colleagues, peers, friends and doctors) as the main source for obtaining information when needed. The latter findings could be attributed to the fact that colleagues are accessible and familiar with the context and are able to provide answers more readily. The findings of this study concur with the findings of other studies conducted by Newman and Doran (2012) in their study on the information-seeking behaviour of professional nurses, as well as that by Murphy *et al.* (2006) in their study on drug information sources used by nurse practitioners and collaborating physicians at the point of care. The latter studies also revealed an inclination for nurses and doctors to seek information from colleagues when needed. Newman and Doran (2012) noted that the information provided by colleagues might not always be accurate, but nurses still trust information from other colleagues because they are accessible at the point of care.

In this study, as in the study conducted by Murphy *et al.* (2006), the professional nurses indicated that printed materials in the form of journals and books were amongst the frequently-used means to access information. Very few of the professional nurses in this study indicated that they made use of electronic sources for accessing information, whereas more than half of the participants indicated that they received health-related information from the radio. The latter findings could be attributed to the fact that, in reality, very few professional nurses have access to electronic sources, whereas almost all of them have access to a radio.

### Ways in which current accessed information is used by professional nurses in practice

According to Ndosi and Newell (2010), nurses need access to reliable evidence-based information sources to allow them to exercise professional judgment in the best interest of their patients. Nurses should use the best available evidence for clinical decision making when providing care to patients. The information accessed by the professional nurses in this study was used mainly for the clinical management of patients (60%), advising patients and communities (57%) and the training of healthcare professionals (42%). Information was also used by the participants for improving their current awareness of health related matters (35%). Less than a quarter of the participants used the information for the public health programmes in which they were involved.

#### Information needed at the point of care by professional nurses

According to Ormandy (2010), an information need is:

[*a*] deficiency in a person's knowledge, a gap in life's experience or a state of uncertainty defined and recognised by the individual, motivating them to seek answers and form questions to find a solution for a particular problem.

Within the health context, an information need is seen as representing a gap or knowledge deficit that could be rectified by information and/or education (Ormandy 2010). The results of this study revealed that 44% of the professional nurses experienced knowledge gaps, whilst 39% indicated that they did not experience any knowledge gaps. The gaps in knowledge experienced by the participants could be because of the fact that many of the participants had completed their nurse training many years ago and did not further their studies. In addition, many of the participants do not have access to information.

According to Newman and Doran (2012), ‘recognition of information needs is important because this cues the nurse to complete patient care tasks with consideration of evidence informed resources’. However, it should be noted that nurses may not perceive or recognise knowledge gaps and do not always seek information when needed.

The four most common topics that the participants indicated as a requirement for accessing information at the point of care included extremely drug-resistant TB (XDR-TB – 76.4%), HIV (72.8%), multi-drug-resistant TB (MDR-TB – 72.1%) and antiretroviral drugs (ARVs – 71.2%). More than half of the participants indicated that information on TB, prevention of mother-to-child transmission of HIV (53%) and non-communicable diseases, such as all the chronic diseases of lifestyle (52.2%), were also a requirement at the point of care. Information on sexually-transmitted infections (43.4%), malaria (42.6%) and public health legislation (40.4%) were also considered a requirement for accessing information at the point of care. Other topics that participants indicated as a requirement for accessing information at the point of care included laboratory services (39.7%), disease surveillance (38.2%), research abstracts on topical subjects in health (38.2%), immunisable diseases (36.8%), diarrhoeal diseases (33.8%), maternal health issues (33.8%) and family planning (29.4%). All the information needs expressed by the professional nurses are evident of South Africa's burden of disease.

The preferred frequency indicated by 52% of the participants for receiving information was monthly and the preferred length for information that they required was three or more printed pages because they needed information that is detailed, but to the point, that they could use for teaching and learning. More than a quarter of the participants indicated that they preferred one printed page of information on the topic that they require as it eliminates unnecessary information and is quick to read and absorb. A few of the participants indicated that the length of the information was not important as long as they could have access to information and that all the information that they require is included in the printed material.

### Practical implications

The research findings were used to make recommendations to stakeholders who utilised the information to develop a mobile library that was loaded onto the storage card of smart phones used by the professional nurses employed in various wards, units and departments for accessing information at the point of care throughout the state hospital where this research was conducted.

## Limitations of the study

There appears to be a paucity of literature pertaining to the information needs of professional nurses and their information-seeking behaviour, especially in South Africa. The findings of the study cannot be generalised to other state hospitals as the sample size was relatively small and non-probability sampling was used.

## Recommendations

In view of the research findings, the following recommen­dations were made:

### Nursing practice

Internet should be introduced into the wards and units so that registered nurses could access information as the need arises. The types of information to be utilised for developing the intervention should include information pertaining to all age groups (from birth to death) and should include:

- Clinical presentation.
- Investigations.
- Treatment.
- Complications, prevention and control.
- Disease management protocols of the Department of Health accessible on their website.
- Access to information should also be enhanced through open education resources and the responsibility of each nurse to engage in Continuing Professional Development (CPD).

### Nursing research

It is hereby recommended that similar research studies be conducted in this field on a wider scale, specifically regarding establishing the extent to which the accessibility to information at the point of care enhances the quality of patient care and clinical nursing practice and patient and staff satisfaction, as there are very few research studies of this nature in South Africa. Comparative studies with our international counterparts could also be explored.

## Conclusion

A quantitative, explorative descriptive survey design was used to conduct this study. It is evident from the research findings that the study successfully achieved its research objectives with regard to exploring and describing the information needs required by professional nurses at the point of care that could enhance nursing practice in the delivery of patient care.

The main results of the study highlighted the types of information that professional nurses had access to and the main sources of information accessed. The ways in which professional nurses utilised the information that they accessed in practice were also revealed. The information needs of professional nurses required at the point of care to enhance nursing practice in the delivery of patient care were identified. The research results were used to develop a mobile library – nurses were trained in its use so that it could be accessed whenever needed.
